# Clinical phenotypes and outcomes of pulmonary hypertension due to left heart disease: Role of the pre-capillary component

**DOI:** 10.1371/journal.pone.0199164

**Published:** 2018-06-19

**Authors:** Sergio Caravita, Andrea Faini, Sandy Carolino D’Araujo, Céline Dewachter, Laura Chomette, Antoine Bondue, Robert Naeije, Gianfranco Parati, Jean-Luc Vachiéry

**Affiliations:** 1 Department of Cardiology, Cliniques Universitaires de Bruxelles, Hôpital Académique Erasme, Bruxelles, Belgium; 2 Department of Cardiovascular, Neural and Metabolic Sciences, Ospedale S. Luca IRCCS Istituto Auxologico Italiano, Milan, Italy; 3 Department of Medicine and Surgery, University of Milano-Bicocca, Milan, Italy; Scuola Superiore Sant'Anna, ITALY

## Abstract

**Background:**

In pulmonary hypertension (PH), both wedge pressure elevation (PAWP) and a precapillary component may affect right ventricular (RV) afterload. These changes may contribute to RV failure and prognosis. We aimed at describing the different haemodynamic phenotypes of patients with PH due to left heart disease (LHD) and at characterizing the impact of pulmonary haemodynamics on RV function and outcome PH-LHD.

**Methods:**

Patients with PH-LHD were compared with treatment-naïve idiopathic/heritable pulmonary arterial hypertension (PAH, n = 35). PH-LHD patients were subdivided in Isolated post-capillary PH (IpcPH: diastolic pressure gradient, DPG<7 mmHg and pulmonary vascular resistance, PVR≤3 WU, n = 37), Combined post- and pre-capillary PH (CpcPH: DPG≥7 mmHg and PVR>3 WU, n = 27), and “intermediate” PH-LHD (either DPG <7 mmHg or PVR ≤3 WU, n = 29).

**Results:**

Despite similar PAWP and cardiac index, haemodynamic severity and prevalence of RV dysfunction increased from IpcPH, to “intermediate” and CpcPH. PVR and DPG (but not compliance, Ca) were linearly correlated with RV dysfunction. CpcPH had worse prognosis (p<0.05) than IpcPH and PAH, but similar to “intermediate” patients. Only NTproBNP and Ca independently predicted survival in PH-LHD.

**Conclusions:**

In PH-LHD, haemodynamic characterization according to DPG and PVR provides important information on disease severity, predisposition to RV failure and prognosis. Patients presenting the CpcPH phenotype appear to have haemodynamic profile closer to PAH but with worse prognosis. In PH-LHD, Ca and NTproBNP were independent predictors of survival.

## Introduction

Post-capillary pulmonary hypertension (PH) is a frequent complication of left heart disease (LHD) [1,2]. However, a small number of PH-LHD patients may present an increase in pulmonary artery pressures (PAP) that cannot be explained by an increase in pulmonary artery wedge pressure (PAWP). In these cases, a cascade of events involving complex interplay of endothelial dysfunction, vasoconstriction and possibly remodeling, may contribute to the development of a pre-capillary component superimposed to post-capillary PH [1–4].

How to define a pre-capillary component in PH-LHD has been debated in recent years. Current ESC/ERS PH guidelines have proposed a haemodynamic definition that subdivides post-capillary PH based on the diastolic pressure gradient (DPG) and on pulmonary vascular resistance (PVR): Isolated post-capillary PH (IpcPH, with PVR ≤3 WU *and/or* DPG <7 mmHg), and Combined post- and pre-capillary PH (CpcPH, with PVR >3 WU *and/or* DPG ≥7 mmHg) [1].

However, this definition has also been recently challenged [5–7]. Firstly, its literal application may result in an ambiguous classification of a proportion of patients (i.e. those with PVR >3 WU *or* DPG ≥7 mmHg, that may fall in both in the IpcPH and in the CpcPH group) [5]. Moreover, each of the two variables (DPG and PVR) presents with certain advantages and disadvantages: the DPG was initially introduced based on solid physiological background as a potential marker of the pre-capillary component [2,3] but its prognostic role is controversial [4, 8–17], while PVR was proposed mainly due to its solid prognostic significance [1,11,18] although it may be highly sensitive to PAWP and cardiac output changes [3]. Finally, pulmonary arterial compliance (Ca) has been suggested to carry additional insights in particular in patients’ description and outcome [17,19–21]. More specifically, Ca has been shown to be sensitive to changes in PAWP in the presence of PH [22]. It has been also suggested to have prognostic significance even in the absence of PH [23].

With the present work, we aimed at describing the clinical and haemodynamic profile of a PH-LHD population assessed in a PH referral center, as compared with patients with pulmonary arterial hypertension (PAH). Secondly, we aimed at evaluating the predictors of prognosis in PH-LHD and determine whether the current classification of IpcPH vs CpcPH provides additional risk stratification.

## Methods

The study had been approved by the Ethics Committee of the Erasme Hospital (ref. n. P2015/359). In agreement with Belgian laws and with local Ethics committee, informed consent was not required according to the retrospective nature of the study. None of the patients had denied the use for research purposes of anonymized data collected in routine clinical practice at an academic hospital.

We retrospectively compared patients with PH undergoing a first elective assessment in stable clinical conditions at the Pulmonary Hypertension and Heart Failure Clinic of the Erasme Hospital between January 2007 and October 2014. Clinical indication for right heart catheterization was given according to international guidelines [1,24]. PH was defined and classified according to recent ESC/ERS guidelines [1]. In particular, PH was defined by a mean PAP ≥ 25 mmHg. Distinction between pre-capillary and post-capillary PH was made based on a PAWP ≤ or > 15 mmHg, respectively [1]. We first compared patients with PH-LHD versus patients with idiopathic or heritable PAH naïf of specific therapy. Then, we subdivided PH-LHD in:

○ IpcPH, if DPG < 7 mmHg and PVR ≤ 3 WU
○ CpcPH, if DPG ≥ 7 mmHg and PVR > 3 WU
○ “intermediate” PH-LHD, if either DPG < 7 mmHg or PVR < 3 WU

We kept for the analysis only patients with complete hemodynamic data and not presenting severe lung disease as a comorbid condition, such as chronic obstructive pulmonary disease (COPD) in Global Initiative for Chronic Obstructive Lung Disease (GOLD) class 4, severe interstitial lung disease. Sleep apnea was not an exclusion criterion. Glomerular filtration rate was calculated according to the Chronic Kidney Disease Epidemiology Collaboration (CKD-EPI) equation [25]. Chronic thromboembolic PH was ruled out in all patients by a combination of clinical history, lung scintigraphy, computed tomography of the chest, pulmonary haemodynamics.

### Haemodynamics

All right heart catheterizations were performed and reviewed by a cardiologist expert in PH. The transducer was zeroed at the midthoracic line in a supine patient, halfway between the anterior sternum and the bed surface [1]. Pulmonary artery pressures were measured at end-expiration and averaged over several cardiac cycles (5 to 8). PAWP was measured by a single expert reader at mid-A wave, as it more closely approximates left ventricular end-diastolic pressure [26]. Cardiac output was measured by thermodilution in triplicate (using an average of three measurements within 10% of agreement). DPG was calculated as the difference between diastolic PAP and PAWP [1–3]. PVR was calculated as (mean PAP–PAWP) / cardiac output [1]. Ca was estimated as the ratio between stroke volume and pulmonary arterial pulse pressure, and the resistance-compliance product (RC-time) as the product of Ca and PVR, and expressed in seconds [22].

### The right ventricle and right ventricular function

RV enlargement was defined as a ratio between right ventricle and left ventricle >1 [1]. Reduced RV systolic function was qualified as a fractional area change ≤ 35% by an experienced cardiologist blinded to invasive haemodynamics. Validation was then performed on a sample of 74 subjects by an independent observer who recalculated fractional area change. RV dysfunction was defined as a combination of reduced RV systolic function and RV enlargement, i.e. as a failing of the homeometric adaptation of the RV faced to an increased afterload [27].

Tricuspid annular plane systolic excursion (TAPSE) was measured according to current recommendations [28,29]. Also, the ratio between TAPSE and systolic PAP was calculated, as a possible surrogate index of RV to pulmonary artery coupling [8].

RV stroke work index (RVSWI), an index of RV workload, was calculated as (mean PAP–right atrial pressure) * (cardiac index / heart rate) * 0.0136.

The ratio between right atrial pressure and PAWP was used as a haemodynamic surrogate of RV dysfunction [30].

### Statistics

Descriptive data are reported as means±standard deviations for continuous variable and as absolute numbers and percentages for categorical variables. Distribution of variables in terms of proximity to normal curve and the homogeneity of variances were detected by Shapiro-Wilk test and Bartlett test, respectively. When needed, either a logarithmic or box-cox transformation was performed to achieve normal distribution. ANOVA with contrasts *a posteriori* was used for variables normally distributed and homoscedastic; otherwise ANOVA on ranks was performed. For multiple comparisons, the algorithm which controls the expected rate of false-positive results for all positive results (false discovery rate) was used. Linear dependence between prevalence of RV dysfunction and TAPSE/systolic PAP on one side, and PVR, DPG and Ca on the other side, was evaluated with Pearson correlation coefficient. Pearson's Chi-squared contingency table test or Fisher Exact test for count data has been used to analyze categorical variables, as necessary.

Estimates of the survival probability were calculated using the Kaplan-Meier method, and the log rank test was employed to test the null hypothesis of equality in overall survival among groups. Univariate and multivariate Cox regression models were calculated to examine factors associated with adverse outcomes. Predictors in the multiple Cox model were selected from the set of variables that reached statistical significance in univariate analysis, by a stepwise procedure with the significance limit set to 0.05, taking care to avoid the simultaneous inclusion of collinear variables. The proportional-hazards assumption was tested using Schoelfeld residuals, with p<0.05 evidence for non-proportionality. Results were expressed as hazard ratios with 95% confidence intervals.

An α level of 0.05 was used for all hypothesis tests. All data analyses were performed using R Core Team (2016), Vienna, Austria.

## Results

From January 2007 to October 2014, 548 patients underwent right heart catheterization. Patients with group 3–5 PH, associated PAH, congenital heart disease, veno-occlusive disease, patients with mean PAP < 25 mmHg as well as patients undergoing right heart catheterization in unstable clinical conditions were discarded from analysis. Thus, one hundred and twenty-eight patients met the inclusion criteria, consisting in 93 patients with PH-LHD and 35 idiopathic/heritable PAH (S1 Fig). The subdivision of PH-LHD according to the haemodynamic classification is depicted in Table 1.

**Table 1:** Patients’ subdivision according to the haemodynamic classification of pulmonary hypertension due to left heart disease.

*PH-LHD* *N = 93*		*DPG (mmHg)*	
		< 7	≥ 7
*PVR (WU)*	≤ 3	*IpcPH* *N = 37*	*Intermediate* *N = 1*
	> 3	*Intermediate* *N = 28*	*CpcPH* *N = 27*

CpcPH = combined post- and pre-capillary pulmonary hypertension; DPG = diastolic pressure gradient; IpcPH = isolated post-capillary pulmonary hypertension; PH-LHD = pulmonary hypertension due to left heart disease; PVR = pulmonary vascular resistance.

### General characteristics

Table 2 summarizes the general characteristics of the study population. Pooled together, PH-LHD were heavier than PAH patients, and presented with slightly lower glomerular filtration rate. NYHA class and NTproBNP levels did not differ between the two groups.

**Table 2:** Patients’ general characteristics.

	PAH n = 35	PH-LHD N = 93	CpcPH n = 27	Interm n = 29	IpcPH n = 37	p overall
**Demographics &** **anthropometrics**						
*Females*, *n (%)*	19 (54%)	51 (55%)	14 (52%)	16 (55%)	21 (57%)	0.984
*Age (years)*	57±16	64±13	64±14	67±13	62±13	0.075
*Height (cm)*	165±10	166±10	168±10	166±10	165±9	0.560
*Weight (Kg)*	71±14	79±17	85±16	75±17	79±17	**0.005**
*BMI (Kg/m*^*2*^*)*	26±5	29±5	30±5	27±4	29±6	**0.010**
**NYHA class**						0.372
*I-II*	7 (20%)	29 (31%)	8 (30%)	7 (24%)	14 (38%)	
*III-IV*	28 (80%)	64 (69%)	19 (70%)	22 (76%)	23 (62%)	
**Blood tests**						
*Creatinine (mg/dL)*	1.1±0.4	1.4±0.7	1.4±0.6	1.5±0.9	1.3±0.6	0.148
*eGFR (mL/min/1*.*73m*^*2*^*)*	70±24	59±25	58±25	55±26	63±25	0.056
*Sodium (mmoL/L)*	141±3	141±4	141±3	140±4	141±4	0.829
*NT-proBNP (pg/mL)*	2930±3002	3685±6936	4136±3845	5034±11573	2214±2126	0.211

BMI = body mass index; CpcPH = combined post- and pre-capillary pulmonary hypertension; eGFR = estimated glomerular filtration rate; Interm = intermediate; IpcPH = Isolated post-capillary pulmonary hypertension; NYHA = New York Heart Association; NT-proBNP = N-terminal pro Brain Natriuretic Peptide; PAH = pulmonary arterial hypertension; RV = right ventricle.

When further subdividing PH-LHD in IpcPH, intermediate and CpcPH, general characteristics were similar in the three groups, although CpcPH presented with slightly higher weight and body mass index than intermediate patients (p<0.05).

The underlying causes of PH-LHD were similar between the three groups, with heart failure with preserved ejection fraction being the leading etiology, followed by heart failure with reduced ejection fraction and a small number of patients with valvular heart disease (S1 Table). Patients with heart failure and a reduced ejection fraction were more likely treated with drugs acting on the renin-angiotensin-aldosterone system and with beta-blockers as compared with patients with a preserved ejection fraction (p<0.01), while diuretics prescription was similar among subgroups. Known obstructive sleep apnea was not represented in the “intermediate” group. The remaining spectrum of comorbidities, as well as background treatment, did not differ between the three subgroups (S1 Table). COPD GOLD 2 was diagnosed in 3 PAH, 3 CpcPH, 1 “intermediate”, 1 IpcPH patients, while COPD GOLD 3 only in 2 IpcPH patients.

### Haemodynamics

The hemodynamic profile was more disturbed in PAH patients (higher pulmonary pressures, gradients and PVR, and lower Ca and cardiac index) as compared with PH-LHD (Table 3).

**Table 3:** Patients’ invasive hemodynamic profile.

	PAH n = 35	PH-LHD n = 93	CpcPH n = 27	Interm n = 29	IpcPH n = 37	ANOVA
**HR (bpm)**	79±15	71±13	74±13	71±14	69±13	**0.017**
***Systolic BP***	126±18	130±30	131±31	137±31	123±28	0.183
***Diastolic BP***	79±12	72±14	74±16	74±13	67±11	**0.002**
***Mean BP***	95±12	91±17	94±18	95±17	86±14	**0.037**
***Systolic PAP (mmHg)***	86±17	61±18	76±16	63±16	48±8	**<0.001**
***Diastolic PAP (mmHg)***	37±8	28±8	35±7	28±6	23±4	**<0.001**
***Mean PAP (mmHg)***	53±11	39±10	49±9	39±8	31±5	**<0.001**
**PAWP (mmHg)**	10±4	23±5	24±6	25±6	22±4	**<0.001**
**RAP (mmHg)**	8±5	13±6	14±6	13±6	11±5	**<0.001**
**RAP/PAWP**	0.88±0.46	0.54±0.21	0.60±0.22	0.54±0.21	0.50±0.21	**<0.001**
**SaO2 (%)**	92±4	96±3	94±5	97±3	97±3	**<0.001**
**SvO2 (%)**	59±10	63±8	60±7	64±8	64±8	**0.005**
**Stroke volume (mL)**	45±14	59±19	58±21	54±14	64±20	**<0.001**
**CI (L/min/m**^**2**^**)**	1.9±0.4	2.2±0.6	2.3±0.5	2.1±0.6	2.2±0.7	**0.041**
**TPG (mmHg)**	44±11	16±8	25±8	15±5	10±3	**<0.001**
**DPG (mmHg)**	27±9	5±5	11±5	3±2	1±2	**<0.001**
**PVR (WU)**	13±4	4±3	7±4	4±1	2±1	**<0.001**
**Ca (mL/mmHg)**	0.9±0.3	2.1±1.1	1.5±0.6	1.8±1.3	2.7±0.9	**<0.001**
**RC-time (s)**	0.69±0.13	0.41±0.13	0.51±0.08	0.40±0.16	0.35±0.07	**<0.001**

BP = systemic blood pressure; CI = cardiac index; CpcPH = combined post- and pre-capillary pulmonary hypertension; DPG = diastolic pressure gradient; Interm = intermediate; IpcPH = isolated post-capillary pulmonary hypertension; PAH = pulmonary arterial hypertension; PAP = pulmonary artery pressure; PAWP = pulmonary artery wedge pressure; PVR = pulmonary vascular resistance; RAP = right atrial pressure; RC-time = resistance-compliance product; TPG = transpulmonary pressure gradient; SaO2 = arterial oxygen saturation; SvO2 = mixed venous oxygen saturation.

When further subdividing PH-LHD, we found that the severity of the hemodynamic profile (pulmonary pressures, pulmonary gradients and PVR) stepwisely increased from IpcPH to intermediate to CpcPH to PAH (Table 3). However, diastolic and mean pulmonary pressure did not significantly differ when comparing CpcPH and PAH (p = 0.308 and p = 0.052, respectively), and Ca was similar between CpcPH and intermediate patients (p = 0.169). There was no significant difference in PAWP and cardiac index across the three subgroups of PH-LHD, although PAWP was slightly higher and stroke volume slightly lower in intermediate patients compared with IpcPH (p = 0.053 and p = 0.063, respectively). All patients but one in the intermediate group had PVR > 3 WU and DPG < 7 mmHg. When subdividing patients according to left ventricular ejection fraction, we could not find any difference in the principal haemodynamic variables except in cardiac index, which resulted lower in patients with a reduced than in those with a normal left ventricular ejection fraction (1.9±0.4 vs 2.5±0.6, p<0.01).

### Effect of the DPG on the PVR-Ca relationship

**Fig 1:**
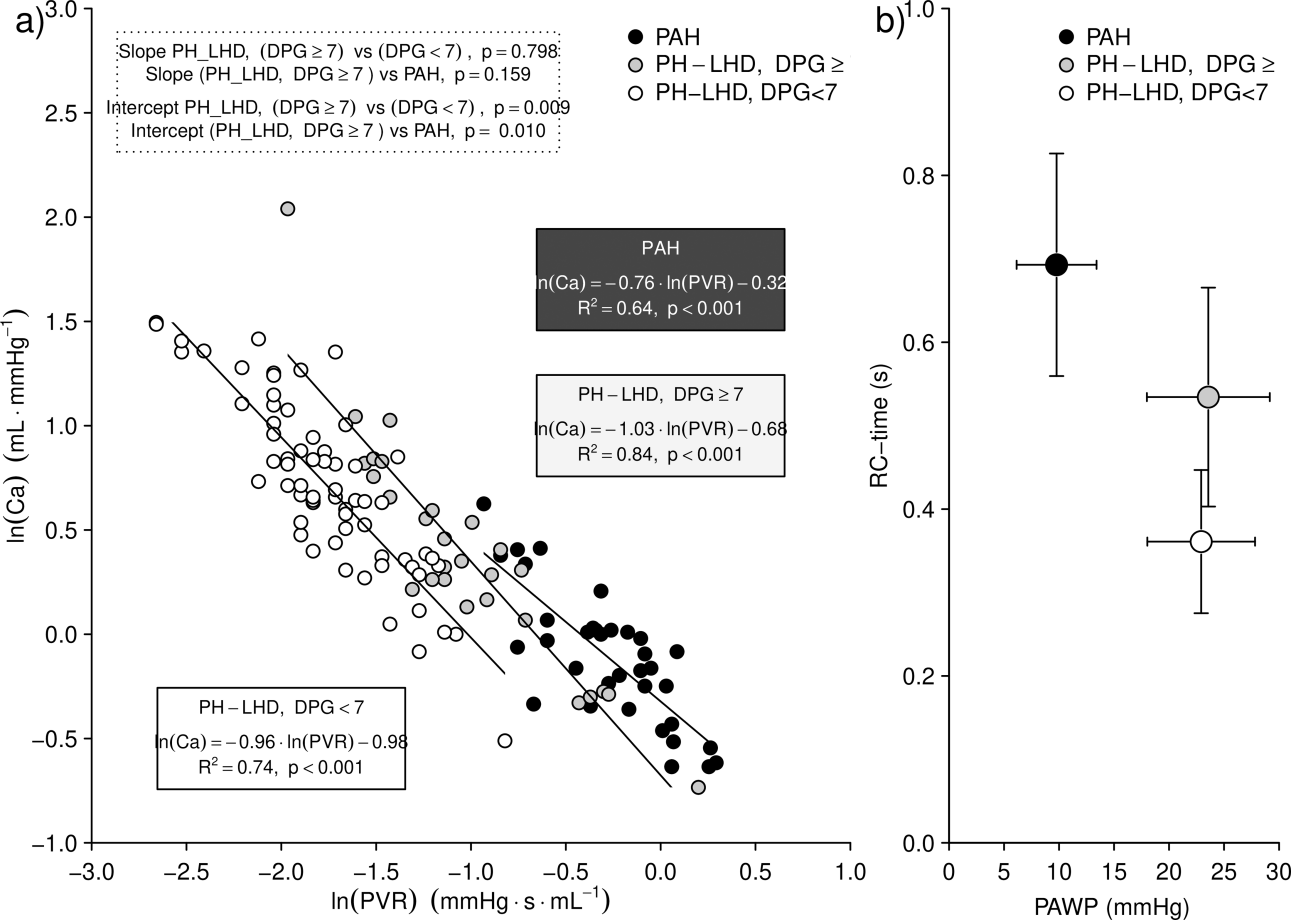
Effects of varying the diastolic pressure gradient and pulmonary artery wedge pressure on the compliance-resistance relationship in patients with pulmonary hypertension. **Panel a): pulmonary arterial compliance as a function of pulmonary vascular resistance, both logarithmically transformed. Panel b): resistance-compliance product as a function of pulmonary artery wedge pressure.** Ca = pulmonary arterial compliance; DPG = diastolic pressure gradient; PAH = pulmonary arterial hypertension; PH-LHD = pulmonary hypertension due to left heart disease; PVR = pulmonary vascular resistance; RC-time = pulmonary vascular resistance-compliance product.

Fig 1A depicts the Log(PVR)–Log(Ca) as a function of PAWP and DPG. PH-LHD with low DPG, PH-LHD with high DPG and PAH all presented with a similar slope of the Log(PVR)-Log(Ca) regression relationship, but with different intercepts, so that the slope of PH-LHD with high DPG lied in between PH-LHD with low DPG and PAH. The relation between RC-time and PAWP across the three different conditions was not linear as it would have been expected, with PH-LHD with high DPG presenting with higher RC-time than PH-LHD with a low DPG for a nearly identical PAWP (Fig 1B).

### RV afterload and RV dysfunction

We kept in the analysis only the echocardiographic examinations which had been performed between 90 days before and one week after right heart catheterization, given that there were no significant changes in treatment between echocardiography and invasive haemodynamic assessment. Eighty-three percent of echocardiography had been performed within 72 hours from right heart catheterization, 91% within 1 week and 97% between 1 month and a few days after right heart catheterization. Mean and median time span between right heart catheterization and echocardiography was -4 ±12 days and -1 (-78 to +6) days, respectively. Thus, a minimal data set from echocardiography was available in 119 over 128 patients.

RV enlargement was more prevalent in PAH than in PH-LHD patients. Moreover, RV enlargement became progressively more frequent passing from IpcPH to “intermediate” PH-LHD to CpcPH to PAH (Table 4).

**Table 4:** Right ventricular function in the different haemodynamic subgroups.

	PAH	PH-LHD	CpcPH	Interm	IpcPH	p overall
*RVOT*, *proximal (mm)*	37±6	32±6	36±7	30±6	31±6	**<0.001**
*RV/LV≥1*	82%	51%	69%	39%	46%	**0.001**
*Reduced RV systolic function*	79%	47%	52%	45%	38%	**0.005**
*TAPSE (mm)*	15±4	18±6	18±5	17±5	19±7	0.083
*TAPSE/sPAP (mm/mmHg)*	0.18±0.07	0.32±0.14	0.25±0.08	0.29±0.11	0.40±0.16	**<0.001**
*RVSWI (g/m*^*2*^*/beat)*	15.6±5.3	11.3±5.1	14.1±5.8	10.7±4.8	9.8±4.1	**<0.001**

CpcPH = combined post- and pre-capillary pulmonary hypertension; Intem = “intermediate PH-LHD”; IpcPH = isolated post-capillary pulmonary hypertension; LV = left ventricle; PAH = pulmonary arterial hypertension; PH-LHD = pulmonary hypertension due to left heart disease; sPAP = systolic pulmonary artery pressure; RV = right ventricle; RVOT = right ventricular outflow tract; RVSWI = right ventricular stroke work index; TAPSE = tricuspid annular plane systolic excursion.

**Fig 2:**
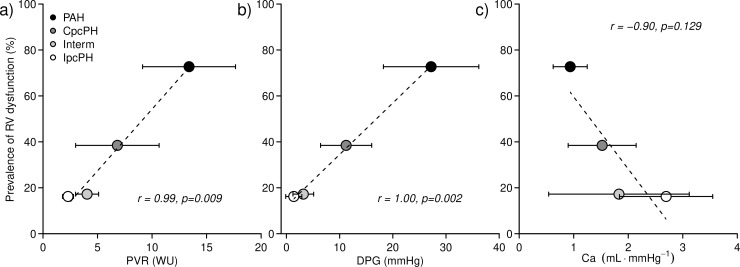
**Prevalence of echocardiographic signs of right ventricular dysfunction in the four groups of patients as a function of pulmonary haemodynamics: pulmonary vascular resistance (panel a), diastolic pressure gradient (panel b), and pulmonary arterial compliance (panel c).** Ca = pulmonary arterial compliance; CpcPH = combined post- and pre-capillary pulmonary hypertension; DPG = diastolic pressure gradient; Interm = intermediate; IpcPH = isolated post-capillary pulmonary hypertension; PAH = pulmonary arterial hypertension; PVR = pulmonary vascular resistance; RV = right ventricle.

Echocardiographic signs of reduced RV systolic function were more represented in PAH as compared with PH-LHD (Table 4). However, the prevalence RV dysfunction showed a crescendo from IpcPH to “intermediate” to CpcPH to PAH patients (p<0.001). Mean PVR and mean DPG of each hemodynamic subgroup resulted highly correlated with the prevalence of RV dysfunction (Fig 2), while the relation between RV dysfunction and Ca was not linear. A similar pattern of correlation was found between the same haemodynamic variables on one side and the ratio between right atrial pressure and PAWP (S2 Fig).

TAPSE, which was available in 80% of cases, did not significantly differ between groups, while the ratio between TAPSE and systolic PAP progressively decreased from IpcPH to “intermediate” PH-LHD to CpcPH to PAH (Table 4). TAPSE/systolic PAP resulted highly correlated with Ca but not with DPG and PVR (S3 Fig).

### Outcome predictors in PH-LHD

Follow-up data were available for 115 patients (90%). There were 36 deaths (31%) over a median follow up of 26 months (25^th^ and 75^th^ percentile: 11 and 46 months).

**Fig 3:**
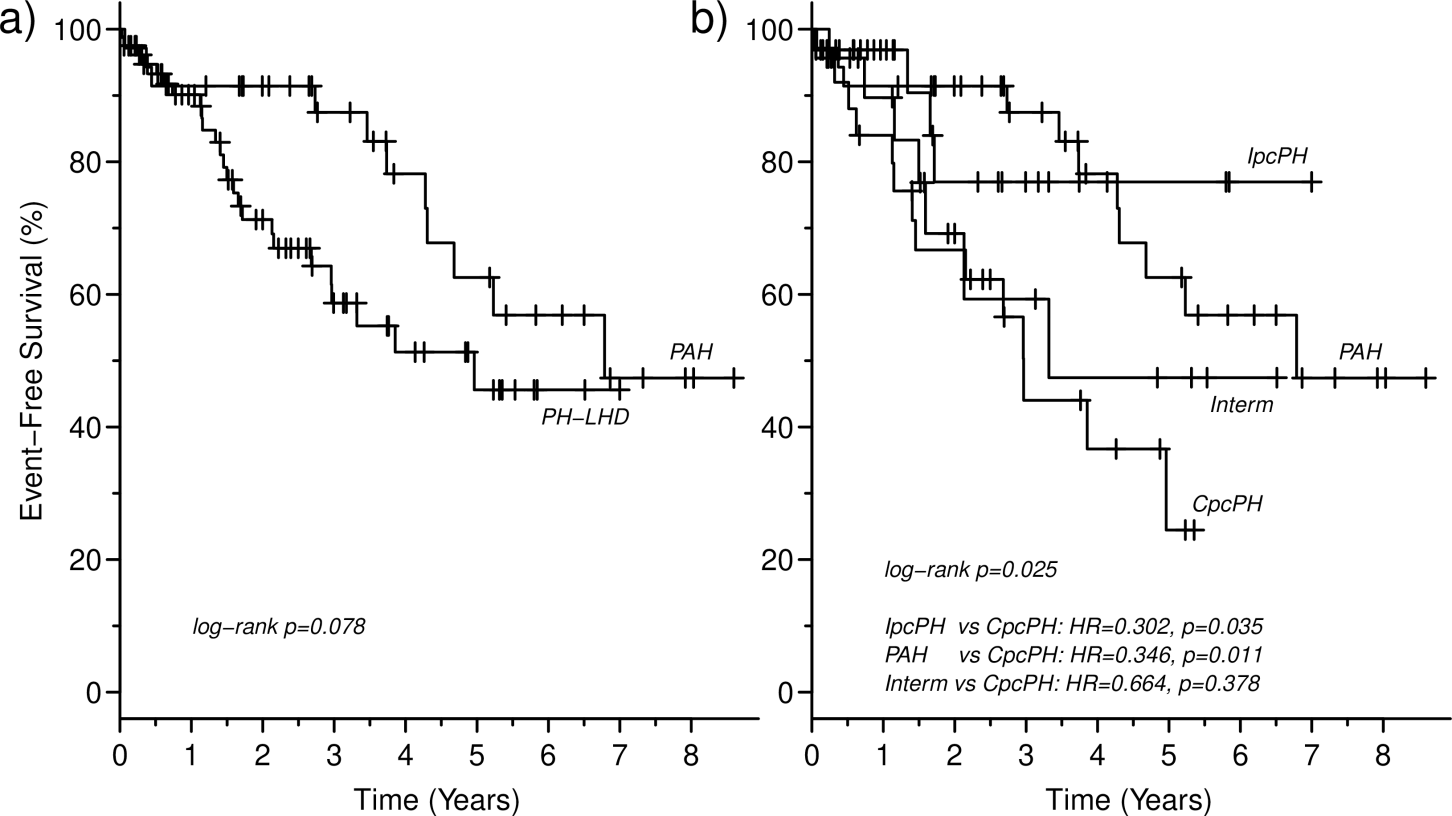
Kaplan-Meier curves of survival for patients with pulmonary arterial hypertension and patients with pulmonary hypertension due to left heart disease. **In the panel a) patients with pulmonary hypertension due to left heart disease are all pooled together, while in the panel b) they are subdivided in three groups according to the diastolic pressure gradient and pulmonary vascular resistance.** CpcPH = combined post- and pre-capillary pulmonary hypertension; Interm = intermediate PH-LHD; IpcPH = isolated post-capillary pulmonary hypertension; PAH = pulmonary arterial hypertension; PH-LHD = pulmonary hypertension due to left heart disease.

At univariate analysis, glomerular filtration rate, NTproBNP, echocardiographic signs of RV dysfunction, diastolic, mean and systolic pulmonary pressures, as well as PVR and Ca were independent predictors of mortality (Table 5). However, at multivariate analysis, only NTproBNP and Ca maintained their prognostic power (Table 5). Survival was similar in PH-LHD and PAH (Fig 3A). However, subgrouping PH-LHD according to DPG and PVR revealed a worse prognosis in CpcPH as compared both with IpcPH and PAH. Survival was similar in CpcPH and in “intermediate” patients (Fig 3B). There were 14 events (56%) in the CpcPH population, 11 events (31%) in the PAH population, 7 events (30%) in the “intermediate” population, and 4 events (13%) in the IpcPH population.

**Table 5:** Univariate and multivariate predictors of survival in pulmonary hypertension due to left heart disease.

	Univariate		Multivariate	
	Hazard Ratio (95% CI)	p-value	Hazard Ratio (95% CI)	p-value
*Clinical parameters*	** **	** **	** **	** **
Age, years	1.016 (0.986–1.046)	0.302		
Male sex	1.501 (0.740–3.047)	0.260		
BMI, Kg/m^2^	0.959 (0.894–1.028)	0.236		
NYHA III-IV	0.772 (0.356–1.672)	0.511		
Ischemic aetiology	0.982 (0.479–2.014)	0.960		
Atrial fibrillation	1.137 (0.437–2.960)	0.793		
Diabetes mellitus	1.504 (0.720–3.139)	0.277		
Smoking history	0.954 (0.474–1.919)	0.895		
Heart rate, beats/min	1.021 (0.994–1.048)	0.134		
*Laboratory parameters*				
Hb, g/dL	0.937 (0.805–1.091)	0.403		
**eGFR, mL/min/1.73 m**^**2**^	**0.979 (0.964–0.993)**	**0.004**	** **	** **
**NTproBNP > 1400 pg/mL**	**4.768 (1.662–13.678)**	**0.004**	**5.066 (1.751–14.660)**	**0.003**
*Echocardiographic variables*				
Reduced LV EF	0.915 (0.435–1.927)	0.816		
**RV dysfunction**	**2.242 (1.095–4.592)**	**0.027**		
**TAPSE/systolic PAP ≤ 0.35**	**1.565 (0.514–4.767)**	**0.430**		
*Haemodynamic variables*				
**Mean PAP, mmHg**	**1.034 (1.003–1.066)**	**0.030**		
**Systolic PAP, mmHg**	**1.018 (1.000–1.037)**	**0.050**	** **	** **
**Diastolic PAP, mmHg**	**1.044 (1.003–1.088)**	**0.036**		
PAWP, mmHg	1.051 (0.984–1.123)	0.136		
RAP, mmHg	1.011 (0.955–1.070)	0.709		
RAP/PAWP	0.706 (0.146–3.399)	0.664		
DPG, mmHg	1.049 (0.993–1.108)	0.088		
TPG, mmHg	1.037 (0.999–1.077)	0.058		
Cardiac Index, L/min/m^2^	0.941 (0.526–1.686)	0.839		
RVSWI, g/m^2^/beat	1.037 (0.975–1.103)	0.246		
**PVR, WU**	**1.130 (1.016–1.256)**	**0.024**	** **	** **
**Ca, mL/mmHg**	**0.602 (0.391–0.926)**	**0.021**	**0.509 (0.298–0.872)**	**0.014**

BMI = body mass index; Ca = pulmonary arterial compliance; DPG = diastolic pressure gradient; eGFR = estimated glomerular filtration rate; Hb = haemoglobin; LV EF = left ventricular ejection fraction; NTproBNP = N terminal pro Brain Natriuretic Peptide; NYHA = New York Heart Association; PAP = pulmonary artery pressure; PAWP = pulmonary artery wedge pressure; PVR = pulmonary vascular resistance; RAP = right atrial pressure; RV = right ventricle; TPG = transpulmonary pressure gradient.

When testing the capability of DPG, TPG, PVR and Ca to predict mortality by ROC curves analysis, we did not find significant differences between the four hemodynamic variables, with area under the curve in between 69 and 75% (S4A Fig). Compared with the optimal cut-off value of ≥3 mmHg, a DPG of ≥7 mmHg was associated with increased (79%) specificity at the expenses of lower (47%) sensitivity (S4B Fig).

## Discussion

In our population of PH, we could confirm that deranged pulmonary haemodynamics is associated with poor survival. However, subdivision of PH-LHD according to DPG and PVR allowed the discrimination of different profiles of disease severity, so that the prevalence of RV dysfunction increased with increasing DPG and PVR. Moreover, we could show the effect of high/low PAWP and high/low DPG on the PVR-Ca relationship in our PH population, demonstrating that patients with PH-LHD and high DPG may display some characteristics of the pulmonary circulation in between IpcPH and PAH. Finally, despite several differences in pulmonary haemodynamics between patients with high DPG and high PVR as compared with patients with high DPG and/or high PVR, prognosis was similar in these two groups, and only Ca and NTproBNP independently predicted outcome in our PH-LHD population.

Our population reflects current knowledge: PH, whichever its cause, is associated with poor functional status (high NYHA class), significant neurohumoral activation (high NT-proBNP levels) and poor outcomes [1,31]. Importantly, the haemodynamic impact of PH seemed to be largely independent of LHD etiology and of background treatment, with few differences in haemodynamic parameters between heart failure with preserved and heart failure with reduced ejection fraction. Moreover, survival of PH-LHD was not different from PAH.

However, further subdividing PH-LHD according to DPG and PVR allowed the discrimination of a subgroup of patients that may present a milder form of PH (namely IpcPH) as opposed to “intermediate” patients and to CpcPH. Indeed, in spite of minimal differences in general patients’ characteristics, the haemodynamic profile of the three PH-LHD subgroups diverged significantly: haemodynamic severity increased stepwisely from IpcPH to “intermediate” to CpcPH, so that this latter resulted closer to PAH. Such haemodynamic differences were somehow paralleled by a stepwise increase in RV dimensions and RV workload, suggesting that they could be maladaptive, negatively impacting on the RV.

Noteworthy, the degree of PAP, pulmonary vascular gradients and PVR elevation resulted largely independent of PAWP when comparing IpcPH and CpcPH, reinforcing the idea that there might be an additional mechanism at pulmonary arterial level that may explain the pre-capillary component in CpcPH [4,16,32]. Interestingly, “intermediate” patients, i.e. predominantly those patients with an isolated elevation of PVR, presented with a slightly higher PAWP and a slightly lower stroke volume than IpcPH, suggesting that in this specific case the increase in PAP and transpulmonary gradient might be partly explained by low flow and by the exaggerated amplification of left-sided filling pressures [3].

In this perspective, our data seem to support the pathophysiological reasoning suggesting that the DPG may be a marker of the pre-capillary component [4,16]. Increasing the DPG in PH-LHD led to a rightward and upward shift of the PVR-Ca relationship, so that patients with PH-LHD and high DPG lied in between PAH and patients with PH-LHD with low DPG, consistently with a previous report [8], and supporting a distinct pulmonary vascular phenotype in CpcPH [16].

Despite this, DPG taken in isolation did not discriminate survival. Haemodynamic predictors of prognosis in LHD may be influenced by the study characteristics (single center vs multicentric), the methodology (univocally standardized haemodynamic assessment with review of single traces vs utilization of protocol data), the characteristics of the referral center (mainly heart failure vs mainly PH center) as well as of the population (heart failure pre-transplant, reduced or preserved left ventricular ejection fraction, valvular heart disease, cardiomyopathies, PH). All these factors may explain at least in part the different and sometimes contrasting results obtained in recent years by several groups [4,8,11,16–18,20,33–37]. In this context, our data seem to confirm that the milder haemodynamic profile of IpcPH (low DPG, low PVR) is mirrored by an overall lower prevalence of RV dysfunction and a clearly overall better prognosis than the other PH-LHD patients [8]. As such, despite several differences in haemodynamics, we may be tempted to pool CpcPH and “intermediate” all together in saying that when patients with PH-LHD have high PVR, their prognosis is dismal [1,11,18] (acknowledging that high DPG with low PVR in PH-LHD appears to be extremely uncommon [5]). In this perspective, the current guidelines subdivision of PH-LHD in two rather than three groups might be sufficient [17]. However, this would neglect that neither PVR nor DPG seem to be independent predictors of outcome in our cohort of PH-LHD. In this perspective, our data suggest that other parameters (both haemodynamic and non-haemodynamic) may better (independently) discriminate prognosis in PH-LHD, in agreement with previous reports [17–20]. In particular, estimates of Ca may be more sensitive to early vascular changes, and accordingly to right heart dysfunction, because of their nonlinear relation with PVR [38], thus confirming its supposed better discriminative potential across patients without PH or with relatively low PVR [10]. Keeping in mind that PVR increased from PH-LHD with low DPG to PH-LHD with high DPG to PAH [31], our data also show that, at a given PVR, Ca would be higher with high than with low DPG, thus linking these three variables and possibly contributing to explain why even some patients with low DPG may present with RV failure and poor outcomes. As such, PVR and DPG should be probably viewed as complementary rather than mutually exclusive, as previously suggested [6,7]. In addition to a hemodynamic phenotype based on these pressure variables, outcome prediction appears reinforced by the integration of Ca [17], parameters of RV function [31] and biomarkers. In other words, a combined approach of a set of variables appears to have more clinical relevance than any one taken in isolation.

Finally, it is also interesting to point out that in a contemporary PH population, newly diagnosed PAH had overall better survival than patients with PH-LHD and a pre-capillary component. This on one side may confirm that approved and currently prescribed treatment in PAH referral centers had impacted the outcome of this condition [1], at the same time reinforcing the need for further research efforts in order to better understand and possibly improve prognosis of the subgroups of PH-LHD at higher risk [39].

### Study limitations

This is a retrospective study done on a relatively small number of patients evaluated at a PH referral center. This selection bias may partly explain differences of our population and of our results with those normally evaluated at mainly heart transplant or heart failure centers. Despite our small sample size, high mortality rates and adequate follow-up time allowed meaningful subgroup analysis.

Ca was not directly measured, but rather estimated by the simplified formula using parameters obtainable from right heart catheterization, acknowledging that this practice, albeit accurate, may result in a significant overestimation of its real value [40]. RV function was evaluated only by means of standard echocardiography and invasive haemodynamics rather than relying on magnetic resonance imaging or 3D echo, both of which allow for better characterization of the complex geometry of the RV.

Finally, we acknowledge that Cheyne-Stokes respiration and central sleep apnea were not routinely assessed, albeit they can be highly prevalent and related both to pulmonary haemodynamics and to prognosis in LHD [41].

## Conclusions

PH-LHD represents a broad spectrum of haemodynamic presentations. The presence of a pre-capillary component, defined by a DPG ≥ 7 mmHg and/or PVR > 3 WU is associated with a worse prognosis as compared with patients with low DPG and low PVR. Ca and NTproBNP may better predict outcome than other clinical and haemodynamic variables suggesting that characterization of patients with PH-LHD should probably incorporate further elements, including RV function. Collaborative study should be encouraged in order to provide a more precise characterization of patients with PH-LHD at high risk, that may help improving the current haemodynamic definition.

## Supporting information

S1 Table Clinical characteristics of patients with pulmonary hypertension secondary to left heart disease. ACE-I = angiotensin converting enzyme inhibitor; ARB = angiotensin receptor blocker; COPD = chronic obstructive pulmonary disease; CpcPH = combined post- and pre-capillary pulmonary hypertension; CRT-D = cardiac resynchronization therapy–defibrillator; HFpEF = heart failure with preserved ejection fraction; HFrEF = heart failure with reduced ejection fraction; Interm = intermediate; IpcPH = isolated post-capillary pulmonary hypertension; ICD = implanted cardioverter defibrillator; O2 = oxygen; OSAS = obstructive sleep apnea syndrome.
(DOCX)

S1 Fig Patients disposition according to the study protocol and haemodynamic definitions. CpcPH = Combined post- and pre-capillary Pulmonary Hypertension; DPG = diastolic pressure gradient; HF = heart failure; IpcPH = Isolated post-capillary Pulmonary Hypertension; LHD = left heart disease; PAH = pulmonary arterial hypertension; PH = pulmonary hypertension; PAWP = pulmonary artery wedge pressure; PVR = pulmonary vascular resistance; RHC = right heart catheterization.
(TIF)

S2 Fig **The ratio of right atrial pressure and pulmonary artery wedge pressure in the four groups of patients as a function of pulmonary haemodynamics: pulmonary vascular resistance (panel a), diastolic pressure gradient (panel b), and pulmonary arterial compliance (panel c).** Ca = pulmonary arterial compliance; CpcPH = combined post- and pre-capillary pulmonary hypertension; DPG = diastolic pressure gradient; Interm = intermediate; IpcPH = isolated post-capillary pulmonary hypertension; PAH = pulmonary arterial hypertension; PAWP = pulmonary artery wedge pressure; PVR = pulmonary vascular resistance; RAP = right atrial pressure.
(PDF)

S3 Fig **The ratio of tricuspid annular plane systolic excursion and systolic pulmonary artery pressure in the four groups of patients as a function of pulmonary haemodynamics: pulmonary vascular resistance (panel a), diastolic pressure gradient (panel b), and pulmonary arterial compliance (panel c).** Ca = pulmonary arterial compliance; CpcPH = combined post- and pre-capillary pulmonary hypertension; DPG = diastolic pressure gradient; Interm = intermediate; IpcPH = isolated post-capillary pulmonary hypertension; PAH = pulmonary arterial hypertension; PAP = pulmonary artery pressure; PVR = pulmonary vascular resistance; TAPSE = tricuspid annular plane systolic excursion.
(PDF)

S4 Fig **Panel a) Receiver operating characteristic curves of haemodynamic predictors of survival in pulmonary hypertension due to left heart disease, with their respective optimal cut-off point, specificity, sensitivity and area under the curve. Panel b) Sensitivity and specificity of different threshold DPG values for predicting outcome.** AUC = area under the curve; Ca = pulmonary arterial compliance; DPG = diastolic pressure gradient; PVR = pulmonary vascular compliance; Sens = sensitivity; Spec = specificity; TPG = transpulmonary pressure gradient.
(PDF)
