# Supplementary material for: Clinical phenotypes and outcomes of pulmonary hypertension due to left heart disease: Role of the pre-capillary component
Source: PLoS One. 2018 Jun 19;13(6):e0199164. doi: 10.1371/journal.pone.0199164 (PMC6007912; doi:10.1371/journal.pone.0199164)
Supplement: S1 Table — ACE-I = angiotensin converting enzyme inhibitor; ARB = angiotensin receptor blocker; COPD = chronic obstructive pulmonary disease; CpcPH = combined post- and pre-capillary pulmonary hypertension; CRT-D = cardiac resynchronization therapy–defibrillator; HFpEF = heart failure with preserved ejection fraction; HFrEF = heart failure with reduced ejection fraction; Interm = intermediate; IpcPH = isolated post-capillary pulmonary hypertension; ICD = implanted cardioverter defibrillator; O2 = oxygen; OSAS = obstructive sleep apnea syndrome. (DOCX) [file pone.0199164.s001.docx]

|  | **IpcPH**  **n=37** | **Interm**  **n=29** | **CpcPH**  **n=27** | **p-value** |
| --- | --- | --- | --- | --- |
| **Etiology of left heart failure** |  |  |  | 0.210 |
| *HFpEF, n (%)* | 19 (51%) | 13 (45%) | 17 (63%) |  |
| *Valvular heart disease, n (%)* | 0 (0%) | 3 (10%) | 2 (7%) |  |
| *HFrEF, n (%)* | 18 (49%) | 13 (45%) | 8 (30%) |  |
| **Comorbidities** |  |  |  |  |
| *Ischemic heart disease, n (%)* | 13 (35%) | 14 (48%) | 5 (19%) | 0.064 |
| *Diabetes Mellitus, n (%)* | 14 (41%) | 10 (35%) | 10 (37%) | 0.956 |
| *Arterial Hypertension, n (%)* | 19 (51%) | 17 (59%) | 18 (67%) | 0.470 |
| *Dyslipidemia, n (%)* | 17 (46%) | 19 (66%) | 11 (41%) | 0.139 |
| *Obesity, n (%)* | 15 (41%) | 8 (28%) | 12 (44%) | 0.384 |
| *Permanent atrial fibrillation, n (%)* | 4 (11%) | 6 (21%) | 4 (15%) | 0.538 |
| *Smoking habits, n (%)* | 16 (43%) | 13 (45%) | 11 (41%) | 0.953 |
| *Chronic obstructive pulmonary disease, n (%)* | 3 (8%) | 1 (3%) | 3 (11%) | 0.380 |
| *Known and treated OSAS, n (%)* | 7 (19%) | 0 (0%) | 6 (22%) | 0.024 |
| **Treatment** |  |  |  |  |
| *Diuretics* | 28 (76%) | 26 (90%) | 22 (82%) | 0.360 |
| *ACE-I or ARBs* | 27 (73%) | 24 (83%) | 19 (70%) | 0.515 |
| *Beta-blockers* | 26 (70%) | 24 (83%) | 19 (70%) | 0.446 |
| *Spironolactone* | 18 (49%) | 12 (41%) | 7 (26%) | 0.182 |
| *Digoxin* | 5 (14%) | 3 (10%) | 4 (15%) | 0.857 |
| *Amiodaron* | 7 (19%) | 7 (24%) | 6 (22%) | 0.872 |
| *Anticoagulant* | 12 (32%) | 13 (45%) | 9 (33%) | 0.536 |
| *Antiplatelet* | 19 (51%) | 12 (41%) | 10 (37%) | 0.491 |
| *Insulin* | 6 (22%) | 7 (24%) | 7 (19%) | 0.872 |
| *O2 therapy* | 4 (15%) | 0 (0%) | 2 (5%) | 0.081 |
| *CRT-D* | 7 (19%) | 5 (17%) | 0 (0%) | 0.363 |
| *ICD* | 5 (13%) | 2 (7%) | 1 (4%) | 0.371 |
