# Supplementary figures and images for: Clinical phenotypes and outcomes of pulmonary hypertension due to left heart disease: Role of the pre-capillary component
Source: PLoS One. 2018 Jun 19;13(6):e0199164. doi: 10.1371/journal.pone.0199164 (PMC6007912; doi:10.1371/journal.pone.0199164)

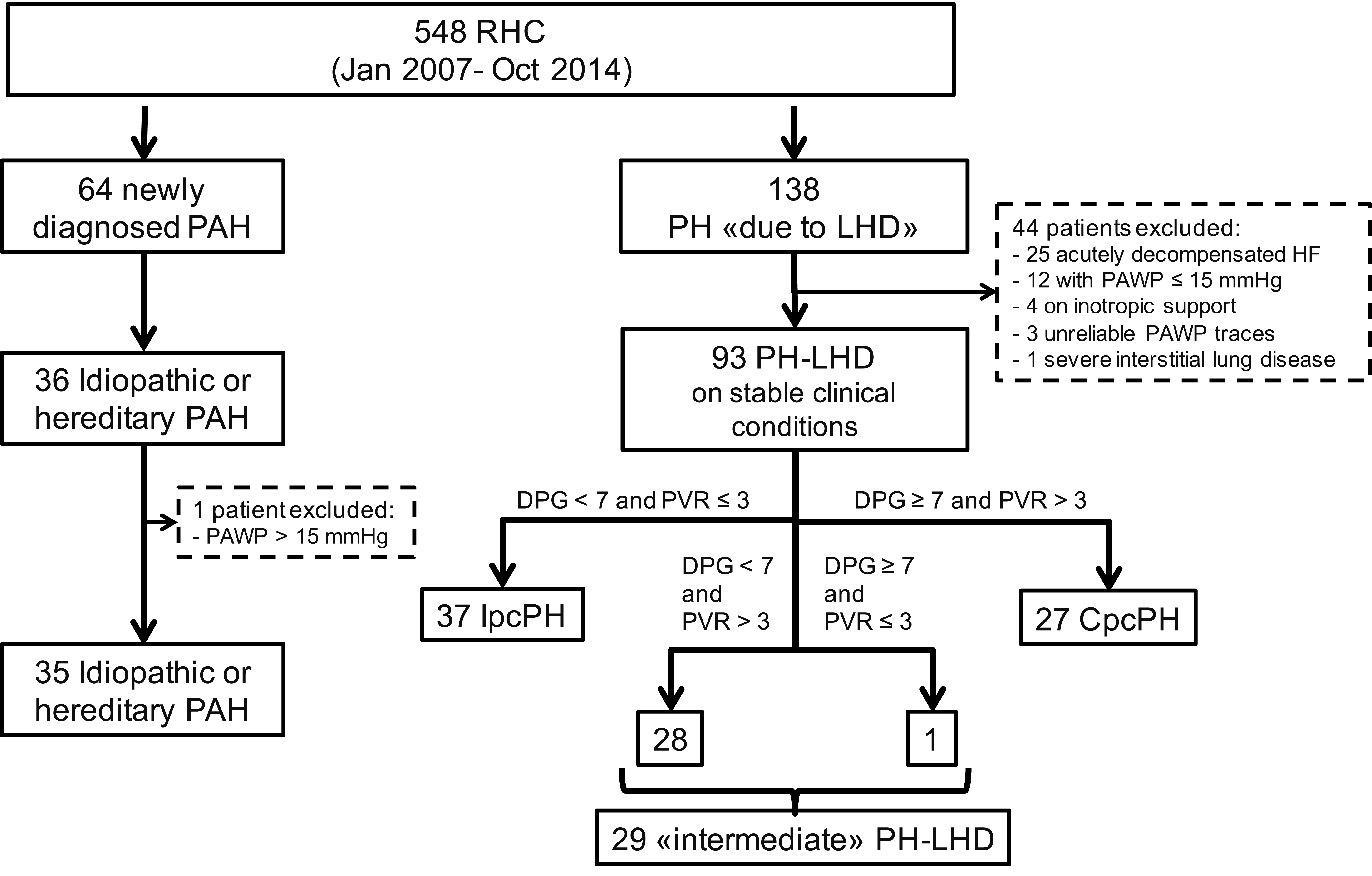

Supplement: S1 Fig — CpcPH = Combined post- and pre-capillary Pulmonary Hypertension; DPG = diastolic pressure gradient; HF = heart failure; IpcPH = Isolated post-capillary Pulmonary Hypertension; LHD = left heart disease; PAH = pulmonary arterial hypertension; PH = pulmonary hypertension; PAWP = pulmonary artery wedge pressure; PVR = pulmonary vascular resistance; RHC = right heart catheterization. (TIF) [file pone.0199164.s002.tif]

a)

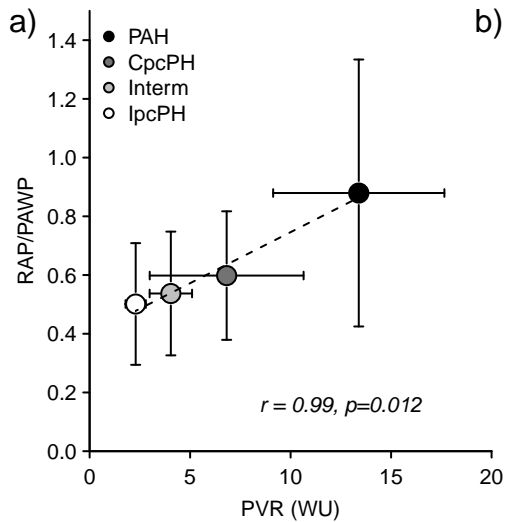

b)

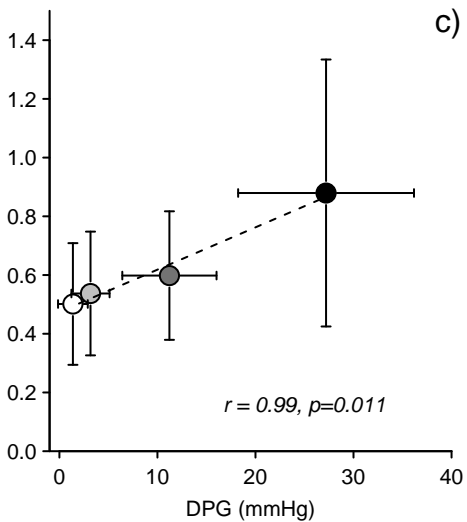

c)

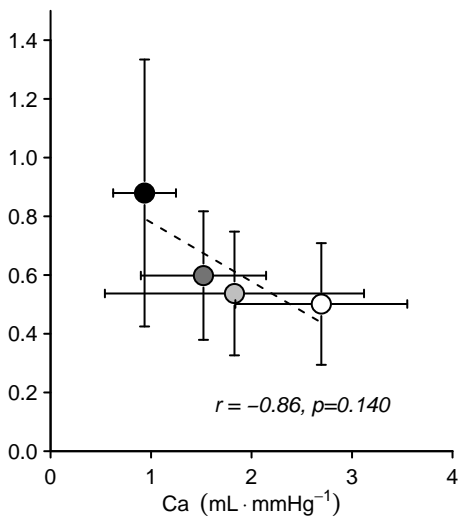

Supplement: S2 Fig — The ratio of right atrial pressure and pulmonary artery wedge pressure in the four groups of patients as a function of pulmonary haemodynamics: pulmonary vascular resistance (panel a), diastolic pressure gradient (panel b), and pulmonary arterial compliance (panel c). Ca = pulmonary arterial compliance; CpcPH = combined post- and pre-capillary pulmonary hypertension; DPG = diastolic pressure gradient; Interm = intermediate; IpcPH = isolated post-capillary pulmonary hypertension; PAH = pulmonary arterial hypertension; PAWP = pulmonary artery wedge pressure; PVR = pulmonary vascular resistance; RAP = right atrial pressure. (PDF) [file pone.0199164.s003.pdf]

a)

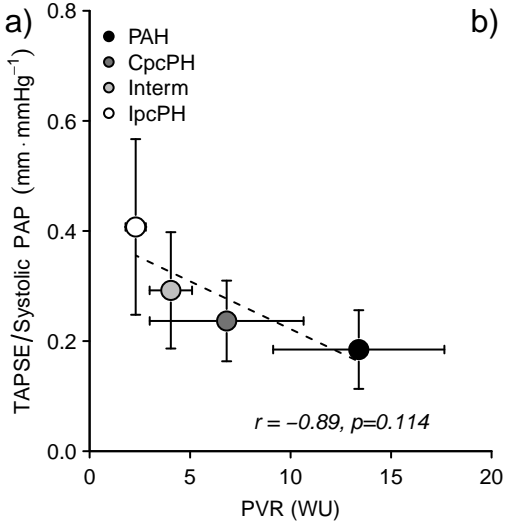

b)

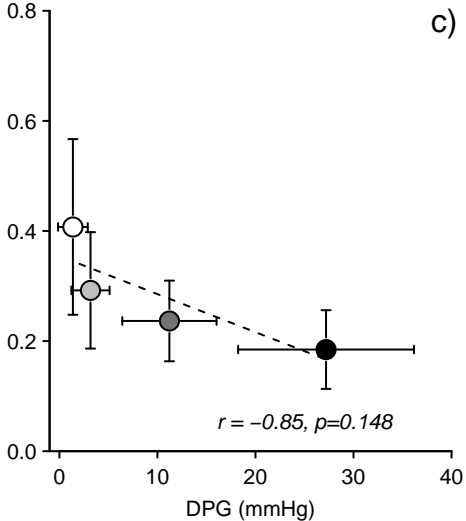

c)

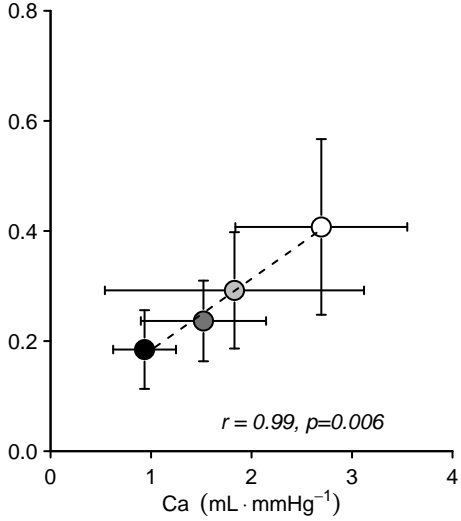

Supplement: S3 Fig — The ratio of tricuspid annular plane systolic excursion and systolic pulmonary artery pressure in the four groups of patients as a function of pulmonary haemodynamics: pulmonary vascular resistance (panel a), diastolic pressure gradient (panel b), and pulmonary arterial compliance (panel c). Ca = pulmonary arterial compliance; CpcPH = combined post- and pre-capillary pulmonary hypertension; DPG = diastolic pressure gradient; Interm = intermediate; IpcPH = isolated post-capillary pulmonary hypertension; PAH = pulmonary arterial hypertension; PAP = pulmonary artery pressure; PVR = pulmonary vascular resistance; TAPSE = tricuspid annular plane systolic excursion. (PDF) [file pone.0199164.s004.pdf]

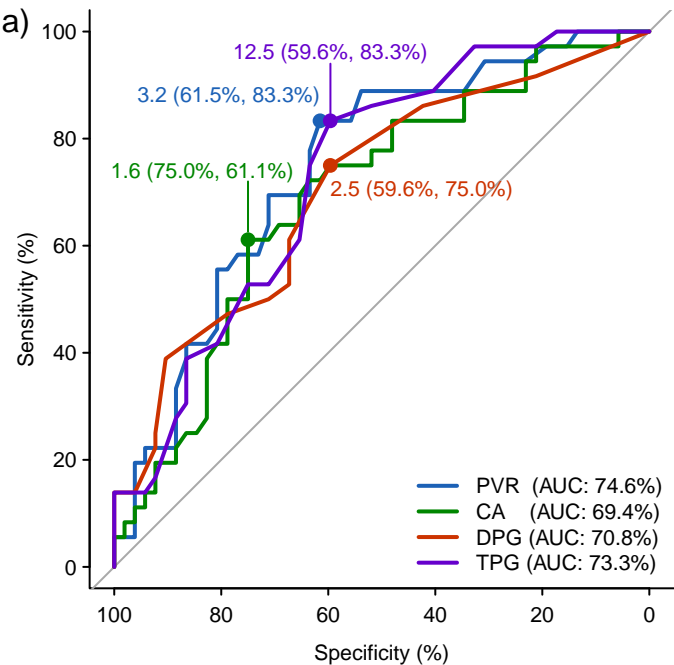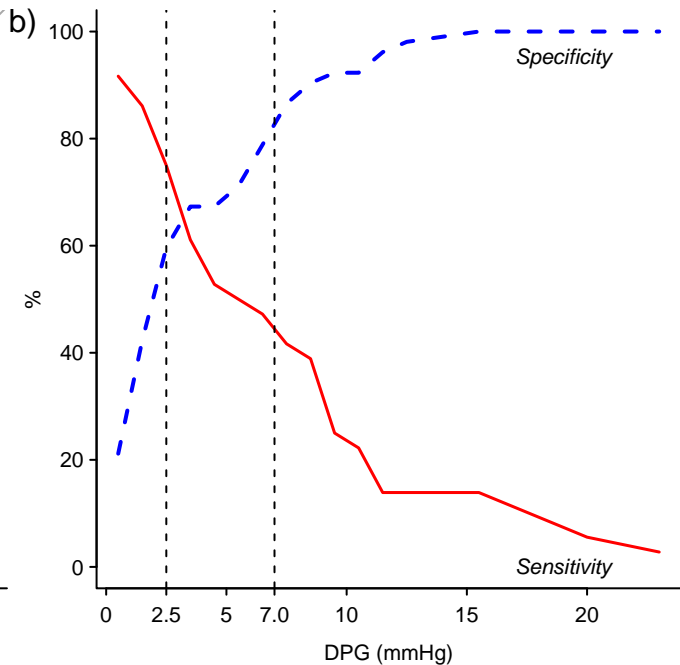

Supplement: S4 Fig — Panel a) Receiver operating characteristic curves of haemodynamic predictors of survival in pulmonary hypertension due to left heart disease, with their respective optimal cut-off point, specificity, sensitivity and area under the curve. Panel b) Sensitivity and specificity of different threshold DPG values for predicting outcome. AUC = area under the curve; Ca = pulmonary arterial compliance; DPG = diastolic pressure gradient; PVR = pulmonary vascular compliance; Sens = sensitivity; Spec = specificity; TPG = transpulmonary pressure gradient. (PDF) [file pone.0199164.s005.pdf]
